# Entomological Surveillance for Zika and Dengue Virus in *Aedes* Mosquitoes: Implications for Vector Control in Thailand

**DOI:** 10.3390/pathogens9060442

**Published:** 2020-06-04

**Authors:** Nathamon Kosoltanapiwat, Jarinee Tongshoob, Preeraya Singkhaimuk, Chanyapat Nitatsukprasert, Silas A. Davidson, Alongkot Ponlawat

**Affiliations:** 1 Department of Microbiology and Immunology, Faculty of Tropical Medicine, Mahidol University, Bangkok 10400, Thailand; nathamon.kos@mahidol.ac.th (N.K.); jarinee.pan@mahidol.ac.th (J.T.); 2 Department of Entomology, Armed Forces Research Institute of Medical Sciences (AFRIMS), Bangkok 10400, Thailand; preerayas.ca@afrims.org (P.S.); chanyapat.nit@gmail.com (C.N.); silas.davidson@westpoint.edu (S.A.D.); 3 Department of Chemistry and Life Science, United States Military Academy West Point, West Point, NY 10996, USA

**Keywords:** zika virus, dengue virus, *Aedes aegypti*, mosquito surveillance, Thailand

## Abstract

Entomological surveillance for arthropod-borne viruses is vital for monitoring vector-borne diseases and informing vector control programs. In this study, we conducted entomological surveillance in Zika virus endemic areas. In Thailand, it is standard protocol to perform mosquito control within 24 h of a reported dengue case. *Aedes* females were collected within 72 h of case reports from villages with recent Zika–human cases in Kamphaeng Phet Province, Thailand in 2017 and 2018. Mosquitoes were bisected into head-thorax and abdomen and then screened for Zika (ZIKV) and dengue (DENV) viruses using real-time RT-PCR. ZIKV RNA was detected in three samples from two female *Ae. aegypti* (1.4%). A partial envelope sequence analysis revealed that the ZIKV sequences were the Asian lineage identical to sequences from ZIKV-infected cases reported in Thailand during 2016 and 2017. Dengue virus-1 (DENV-1) and dengue virus-4 (DENV-4) were found in four *Ae. aegypti* females (2.8%), and partial capsid sequences were nearly identical with DENV-1 and DENV-4 from Thai human cases reported in 2017. Findings in the current study demonstrate the importance of entomological surveillance programs to public health mosquito-borne disease prevention measures and control.

## 1. Introduction

Mosquitoes are vectors of various flaviviruses, which are enveloped, positive-sense single stranded RNA viruses (Family *Flaviviridae*, Genus *Flavivirus*). The most important mosquito transmitted flaviviruses are the dengue (DENV), Zika (ZIKV), yellow fever (YFV), West Nile (WNV) and Japanese encephalitis (JEV) viruses. Among them, DENV, ZIKV and JEV circulate widely in Southeast Asia, including Thailand [1,2,3]. DENV and ZIKV are primarily transmitted to humans by *Aedes* mosquitoes, with *Aedes (Stegomyia) aegypti* serving as the major vector and *Aedes (Stegomyia) albopictus* as a secondary vector; whereas JEV and WNV are predominantly transmitted by *Culex* mosquitoes [1,4,5]. Both dengue and Zika viral infections in humans range from asymptomatic to symptoms of fever, headache, myalgia, arthralgia and maculopapular rash at the initial stage of illnesses [2,6]. DENV infections can be classified as dengue fever (DF), dengue hemorrhagic fever (DHF) or dengue shock syndrome (DSS) according to the severity of symptoms. DHF and DSS can be fatal if appropriate treatments are not provided, making the disease a significant public health concern, especially in endemic areas. Due to the self-limiting nature of the illness and low mortality rates, infection with ZIKV has generally caused less of a significant public health impact than dengue, since its first identification. Although typically milder than infection with DENV, there are two severe ZIKV disease complications of note: Guillain–Barre syndrome and meningoencephalitis. After the Brazilian ZIKV outbreak in 2015, public health scientist noted Guillain–Barre syndrome and a drastic increase in the number of infants with microcephaly born to mothers with the ZIKV infection meningoencephalitis, causing widespread alarm, especially among pregnant women [7,8,9].

DENV has been postulated to have emerged about 1000 years ago in an infectious cycle involving non-human primates and mosquitoes [10]. The first human case of DENV (DENV-1) was isolated in Japan in 1943 [11]. Afterwards, outbreaks of four DENV serotypes (DENV-1, DENV-2, DENV-3 and DENV-4) have been reported globally [12]. As reported by the World Health Organization (WHO), the global incidence of dengue has increased 30-fold during the past five decades, with 50–100 million new infections estimated to occur annually in more than 100 endemic countries. These infections generally occur in tropical and sub-tropical regions [13]. Although more recently, DENV has been reported from previously unaffected, more temperate areas such as France, Croatia, Portugal and other European countries [1]. DENV incidence in Thailand has been reported since the 1950s [12] and several major outbreaks with high morbidity have been documented since then. According to the WHO, during 2004–2010, Thailand was ranked sixth among the 30 most highly dengue-endemic countries in the world [13]. One of the largest DENV epidemics in Thailand was in 1987 with 174,285 cases and 1008 deaths. Recently, more than 72,000 dengue cases with a fatality rate of 0.13% were reported in 2018 to the Ministry of Public Health, Thailand. All four DENV serotypes (DENV-1, DENV-2, DENV-3 and DENV-4) are reported to circulate in Thailand with a recurrent circulation of each serotype [12,14,15]. The Department of Disease Control—part of the Ministry of Public Health (MOPH), Thailand—reported that the major DENV serotypes causing illness in 2017 were DENV-1 and DENV-4. In 2018, DENV-1 remained the dominant serotype and the incidence of DENV-4 decreased.

ZIKV was first isolated from a sentinel rhesus monkey in the Zika forest, Uganda in 1947 and later from a pool of *Ae. africanus* mosquitoes collected in the same forest [16]. Afterwards, serological evidence of ZIKV infections in humans was reported for various African and Asian countries [6,17]. In 1975, Moore et al. reported the first ZIKV isolation from a patient with febrile illness from Nigeria, as well as the isolation of the virus from specimens collected in 1968 [18]. In Southeast Asia, ZIKV circulation was suspected as early as 1954, due to reactivity in a seroprevalence study [5]. Starting in 2009, active ZIKV circulation has been identified in Cambodia [19], Indonesia [20], Malaysia [21], the Philippines [22], Singapore [23], Thailand [2], and Vietnam [24]. The first report of ZIKV in Thailand was in 2013 from reports of infections in Canadian, German and Japanese travelers returning from the Southern part of Thailand [25,26,27]. Later, a retrospective investigation by the Thai MOPH confirmed autochthonous ZIKV cases in several different provinces across Thailand during 2012–2014, indicating wide-spread distribution of ZIKV in the country [2]. ZIKV isolated from this study was from the Asian lineage and is closely related to an isolate from the 2013 French Polynesia outbreak [2]. According to the Bureau of Emerging Infectious Diseases, Department of Disease Control, Thai MOPH, in 2016 and 2017, more than 1600 confirmed ZIKV-infected cases were reported from different regions across the central, north, east and west of Thailand [28].

Although many human cases of ZIKV and DENV infections have been reported in Thailand, there are few reports of ZIKV circulation in wild-caught mosquitoes [29]. In this study, entomological surveillance for arthropod-borne viruses was conducted in Kampheang Phet (KPP) Province, Thailand, a dengue-endemic area where human Zika cases had been recently reported. *Aedes* mosquitoes were screened for ZIKV and DENV and the virus sequences were analyzed. Findings from this study can be used as the critical first step toward developing a routine entomological surveillance and vector control program in Thailand.

## 2. Results

### 2.1. Mosquito Collection

Over 86 trap-days, 488 mosquitoes belonging to four genera, *Aedes, Culex*, *Armigeres* and *Anopheles,* were captured (Table 1). The majority (40.8%) of mosquitoes collected inside houses belonged to the genus *Aedes* with 187 *Ae. aegypti* (130 females and 57 males) and 12 *Ae. albopictus* (11 females and one male).

### 2.2. Virus Detection

A total of 282 samples from 141 *Aedes* females (130 *Ae. aegypti* and 11 *Ae. albopictus*) were screened for ZIKV and DENV. Each female mosquito was separated in two parts (head-thorax and abdomen) to determine virus dissemination. Both samples were tested from each mosquito. ZIKV and DENV RNA was detected only in *Ae. aegypti* collected from houses that had recent human cases of ZIKV. No ZIKV or DENV RNA was detected in *Ae. aegypti* from surveyed households within a 50 m radius from detected human cases. A TaqMan^®^ real-time RT-PCR and ZDC multiplex real-time RT-PCR assay detected a ZIKV genome in three samples (R012, R013 and R015) from two *Ae. aegypti* females (1.4%, 2/141). A partial ZIKV envelope gene was amplified from two samples from the same mosquito (R012, head-thorax and R013, abdomen) by a conventional RT-PCR. DNA sequencing was performed and analyzed by a BLAST search; sequences were identified as ZIKV and shared 100% sequence identity. Four *Aedes* females (2.8%, 4/141) were DENV positive by real-time RT-PCR. Analysis of partial capsid/prM sequences identified three DENV-1 (R248, R251 and R290) and one DENV-4 (R097). The DNA sequences obtained from this study were submitted to the NCBI database and the accession numbers were assigned as MN527290 for R012 (ZIKV), and MN527286, MN527287, MN527288 and MN527289 for R097, R248, R251 and R290 (DENV), respectively.

### 2.3. Phylogenetic Relationship of ZIKV

**Figure 1:**
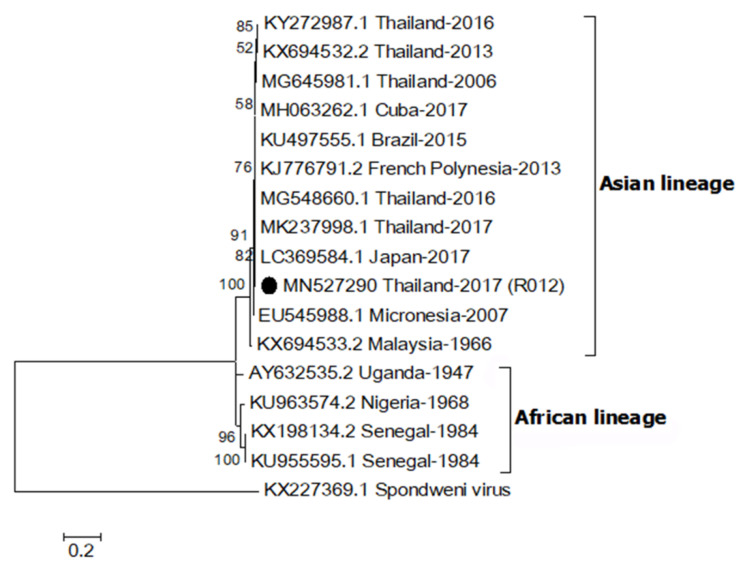
Maximum likelihood phylogenetic tree of Zika virus (ZIKV) partial envelope gene (331 bp). The E gene sequences of ZIKV (R012) found in this study are indicated with black circles. Accession numbers of all reference sequences are provided in the phylogenetic tree. Bootstrap values > 50% are indicated at nodes. A scale bar represents nucleotide substitutions per site. A sequence of Spondweni virus was used as an outlier.

A partial envelope sequence of R012 (MN527290) was compared with other ZIKV strains using multiple sequence alignment. The genetic relationship was analyzed by construction of a phylogenetic tree (Figure 1). The result showed that the R012 sequence belonged to the Asian lineage ZIKV clade (nucleotide sequence identity ≥ 96%) and the alignment is shown in Supplementary Figure S1. In addition, the E gene sequence was 100% identical to the sequence from a ZIKV detected in a citizen from Thailand, reported in 2016 (MG548660.1), from patient serum collected in 2017 from Thailand (MK237998.1), and the sequence from a ZIKV-infected Japanese tourist, who visited Thailand in 2017 (LC369584.1) [30].

### 2.4. Phylogenetic Relationship of DENV

**Figure 2:**
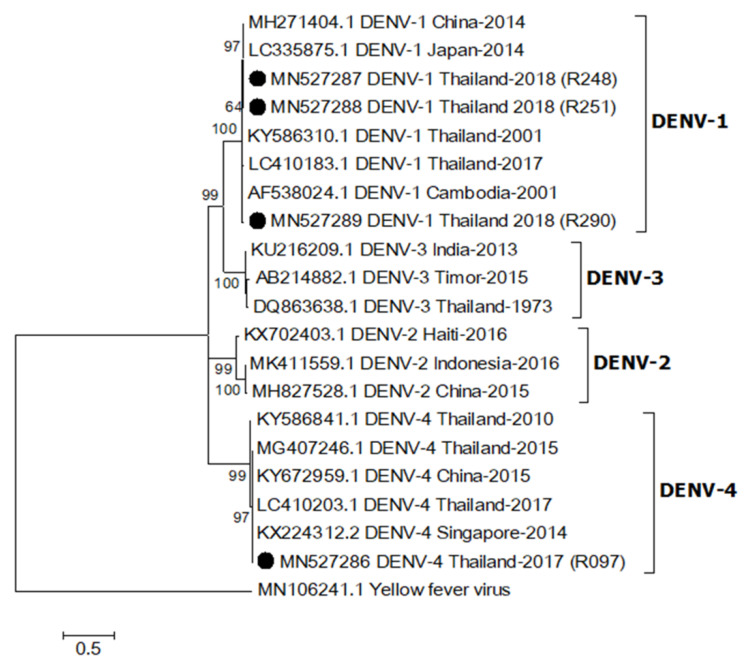
Maximum likelihood phylogenetic tree of dengue virus (DENV) partial capsid/prM gene (395 bp). All DENV capsid/prM sequences found in this study were indicated with black circles. All accession numbers of reference sequences are provided in the phylogenetic tree. Bootstrap values > 50 are indicated at nodes. A scale bar represents nucleotide substitutions per site. Yellow fever virus was used as an outlier.

One sequence from an *Ae. aegypti* collected in 2017 (MN527286) was identified as DENV-4 based on sequence analysis of a partial DENV capsid/prM (nucleotide sequence identity > 98%), whereas the other three sequences from *Ae. aegypti* females caught in 2018 (MN527287-MN527289) were DENV-1 (nucleotide sequence identity > 96%, Figure 2). They were closely related to DENV-1 (LC410183.1) and DENV-4 (LC410203.1) detected in patients from Thailand reported in 2017 [14]. The sequence alignment is shown in Supplementary Figure S2.

## 3. Discussion

Mosquito-based ZIKV and DENV surveillance in KPP was conducted in areas where Zika–human cases were reported recently. Among the four endophilic mosquito genera caught, the majority were *Aedes* mosquitoes (40.8%). Only female *Ae. aegypti* and *Ae. albopictus* were subsequently tested for ZIKV and DENV, as they are considered the major vectors to transmit these viruses. A total of 488 mosquitoes were captured from 86 trap-days (5.7 mosquitoes/trap). This is considerably high since mosquito collections were performed within 72 h after receiving information about human cases of ZIKV from the local hospitals. Local regulations required all houses with confirmed DENV or ZIKV cases to be sprayed with an adulticide within this time period. Surprisingly, both ZIKV and DENV infected mosquitoes were found in adulticide sprayed houses (index houses).

ZIKV was detected in two *Ae. aegypti* samples from the same mosquito specimen collected from a Zika index house. Based on a sequence analysis of the E gene, which has been used to classify ZIKV into African and Asian lineages, the ZIKV detected in this study was of the Asian lineage [31]. Analysis showed the ZIKV sequence was 100% identical to previous human cases of ZIKV reported in Thailand during 2016–2017 [30]. This confirms that the same ZIKV lineage was still circulating in Thailand. The Asian lineage is presently responsible for various global outbreaks, including outbreaks in Thailand [32]. No virus was detected in *Ae. albopictus* specimens but this may be due to the low number of specimens (*n* = 11) collected in this study. Since the mosquito traps were set inside houses, it was not surprising that *Ae. albopictus* was collected less frequently since it is more exophilic than *Ae. aegypti* [5]. In a recent report from Thailand, both *Ae. aegypti* and *Ae. albopictus* were screened for ZIKV and ZIKV was detected in female, male and larvae of field-caught *Ae. aegypti* collected around active ZIKV patients’ houses [29]. That study also did not detect ZIKV in *Ae. albopictus.*

Additionally, we detected DENV in mosquitoes collected from ZIKV patient homes, although no DENV outbreak or dengue cases were reported in the study area during this period. It is possible that the mosquitoes may have acquired DENV from unreported, asymptomatic infected humans, or by vertical transmission [33]. The two DENV serotypes, DENV-1 and DENV-4, found in this study were identical (more than 96% identity) to DENV from human cases reported in 2017 [14], which suggests a connection to an outbreak of those DENV serotypes in Thailand during that time.

This study demonstrates the importance of entomological surveillance for public health programs and to provide guidance to mosquito-borne disease prevention and control. The entomological surveillance is crucial for the planning, implementation, and monitoring of vector control programs. Both adult and larval control measures should be conducted focusing on index houses which can serve as major hubs for transmission of the virus during an outbreak. In this study, disease vectors and infected mosquitoes were captured in index houses although vector control, including adulticide and larvicide application, was performed in homes within 24 h after cases of human disease were confirmed. Therefore, a surveillance system for efficacy of vector control operations (e.g., mosquito sentinel, larval bioassay, etc.) is warranted. This system will provide a warning to local public health officers regarding the effectiveness of the current vector control program.

## 4. Conclusions

The presence of ZIKV and DENV in *Ae. aegypti* mosquitoes collected from houses recently sprayed with adulticide has implications for public health and vector control measures in areas at risk for flavivirus transmission. Since there are no effective vaccines or specific therapeutic treatments for DENV and ZIKV infections, vector control is the only effective tool to mitigate disease transmission. Vector surveillance before and after vector control is essential to validate the efficacy of the control measures employed. Our study suggests that a single application of non-residual adulticide alone may not be sufficient to reduce the risk of further spread of ZIKV by controlling the *Aedes* vector. Routine entomological surveillance is essential for the planning and implementation of an effective vector control program.

## 5. Materials and Methods

### 5.1. Study Site

**Figure 3:**
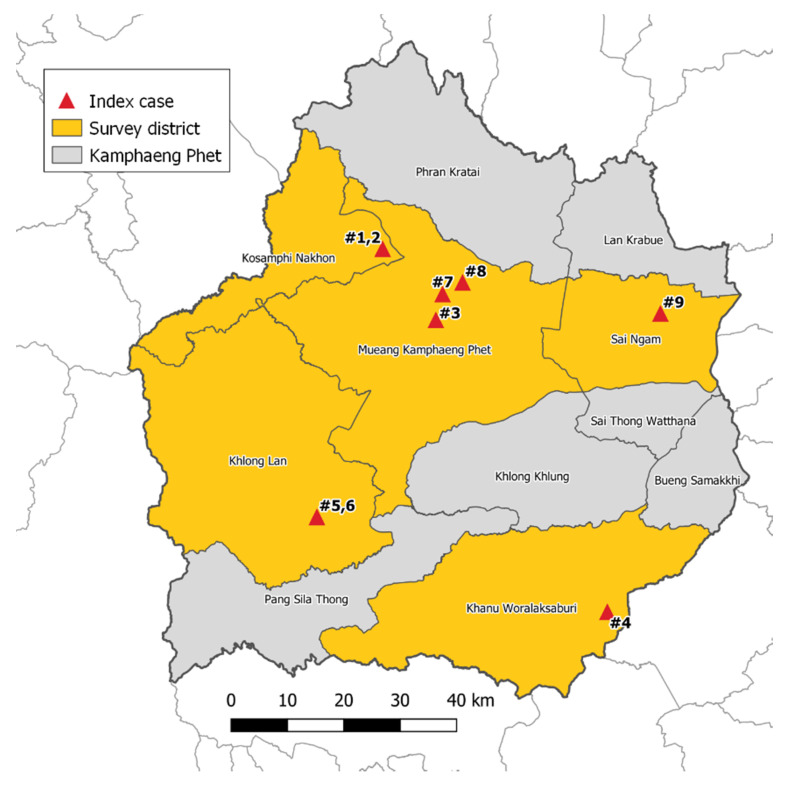
Mosquito collections from 9 Zika index case houses and 25 neighboring houses (within a 50 m radius from the Zika index cases) located in 5 districts, Kamphaeng Phet Province, Thailand during July 2017–February 2018.

From July 2017 to February 2018, mosquito surveillance was performed in 5 districts in KPP, which is located approximately 360 km northeast of Bangkok. The climate in KPP is tropical with three seasons: rainy (June to October), winter (November to February), and summer (March to May). These five districts include Khanu Woralaksaburi, Khlong Lan, Kosamphi Nakhon, Mueang, and Sai Ngam (Figure 3). These are DENV endemic areas but have recently reported human cases of ZIKV. During the study period, there were nine human cases of ZIKV in these study villages reported to the Provincial Public Health Office. Almost all Zika cases (8/9 cases) were reported in the rainy season.

### 5.2. Mosquito Collection

BG Sentinel traps (Biogents AG, Regensburg, Germany) baited with 1 kg of dry ice and BG-lure containing ammonia, lactic acid and caproic acid, were used to collect mosquitoes. Traps were placed inside 34 houses, including houses with ZIKV reported cases (*n* = 9) and neighboring houses within a 50 m radius of Zika index houses (*n* = 25). Based on the feeding behavior of *Aedes* mosquitoes, traps were continuously operated to collect daytime biting mosquitoes for 8 h (0800–1600). Mosquito collection was conducted no longer than 72 h after receiving information about human cases of ZIKV from the local hospitals. Mosquito samples in each trap were stored in a cooler containing dry ice for transportation to the Armed Forces Research Institute of Medical Sciences (AFRIMS) Entomology laboratory in Bangkok, where the samples were further processed. All collected mosquitoes were morphologically identified to species under the stereomicroscope [34]. They were sorted by sex and location prior to keeping in a −80 °C freezer until use.

### 5.3. Detection of ZIKV and DENV by Real-Time RT-PCR

Both *Ae. aegypti* and *Ae. albopictus* females were screened for ZIKV and DENV by real-time RT-PCR. *Aedes* head-thorax parts were dissected from the abdominal parts and separately transferred into microfuge tubes containing RPMI medium. Both mosquito parts (head-thorax and abdomen) were homogenized in RPMI medium using a Bullet Blender^®^ Storm (Next Advance, Inc., Troy, NY, USA). Supernatant was collected for a total nucleic acid extraction using a PureLinkTM Viral RNA/DNA Mini Kit (Thermo Fisher Scientific, Waltham, MA, USA) according to the manufacturer’s instruction.

A TaqMan^®^ Fast Virus 1-Step Master Mix (Applied Biosystems, Foster City, CA, USA) and specific primers and probes were used in the real-time RT-PCR for detection of ZIKV [35] as shown in Table 2. The real-time RT-PCR reaction contained 1× TaqMan^®^ Fast Virus 1-Step Master Mix, 0.5 µM each of forward (ZIKV835 or ZIKV1086) and reverse (ZIKV911c or ZIKV1162c) primers, 0.125 µM probe (ZIKV860-FAM or ZIKV1107-FAM), and 5 µL of the total RNA in a final volume of 20 µL. Thermal cycling conditions were: 50 °C for 15 min, 95 °C for 30 s, followed by 40 cycles of 95 °C for 10 s and 60 °C for 30 s in a CFX96TM Real-Time System (Bio-Rad, Hercules, CA, USA).

The real-time RT-PCR detection of ZIKV and DENV was done using a ZDC Multiplex RT-PCR Assay (Bio-Rad) according to the manufacturer’s instruction. The real-time RT-PCR reaction contained 1× iTaq^TM^ Universal Probes One-Step Reaction Mix, 0.6 μL of iScript^TM^ Reverse Transcriptase, 1× ZDC Multiplex PCR Assay Mix and 5 μL of the total nucleic acid in a final volume of 25 μL. The thermal cycler (CFX96TM Real-Time System) with conditions of 50 °C for 15 min, 94 °C for 2 min, followed by 45 cycles of 94 °C for 15 s, 55 °C for 40 s and 68 °C for 30 s was set.

### 5.4. DNA Sequencing and Phylogenetic Analysis

For nucleotide sequencing, the total RNA extracted from the ZIKV and DENV real-time RT-PCR positive samples were subjected to the RT-PCR with sequencing primers (Table 2) using a SuperScript^®^ III One-Step RT-PCR System with a Platinum^®^ Taq DNA Polymerase kit (Invitrogen, Carlsbad, CA, Bio-Rad). The reaction mixture contained 1× reaction mix, 0.4 μM of each forward (ZIKENVF or D1) and reverse primer (ZIKENVR or D2), 1 μL of SuperScript^®^ III RT/Platinum^®^ Taq Mix, and 5 μL of the total RNA in a final volume of 25 μL. The thermal cycling conditions were set as a reverse transcription at 50 °C for 30 min, followed by an initial denaturation of cDNA at 94 °C for 2 min, 40 cycles of denaturation at 94 °C for 15 s, annealing at 54 °C for 30 s, and an extension at 68 °C for 45 s, followed by a final elongation step at 68 °C for 10 min. The PCR products of 365 bp and 511 bp, for ZIKV and DENV, respectively, were resolved on a 1.5% agarose gel, stained with ethidium bromide, and visualized under a gel documentation system (Gel Doc^TM^ XR+ imaging system, Molecular Imager^®^, Bio-Rad). The PCR products were extracted from gel using the QIAquick gel extraction kit (Qiagen, Hilden, Germany) following the manufacturer’s instructions. Purified DNA was sequenced using the forward and reverse primers. Sequencing chromatograms were inspected and processed using BioEdit 7.0.4.1. [36]. Nucleotide sequences were queried against the NCBI database using BLAST and aligned with published reference sequences using ClustalW in BioEdit. Phylogenetic trees were constructed using the Molecular Evolutionary Genetics Analysis software (MEGA, version 7.0.21) [37]. The maximum likelihood method was applied based on the phylogenetic model analysis. Bootstrap resampling analysis of 1000 replicates was used.

## Figures and Tables

**Table 1:** Total number of collected mosquitoes in Kampheang Phet between July 2017 and February 2018.

Species	Female	Male	Total
*Ae. aegypti*	130	57	187
*Ae. albopictus*	11	1	12
*An. peditaeniatus*	1	-	1
*An. tessellatus*	1	-	1
*An.* sp.	1	-	1
*Ar. subalbatus*	4	-	4
*Cx. brevipalpis*	3	-	3
*Cx. quinquefasciatus*	62	123	185
Cx. *vishnui*	88	5	93
*Cx.* sp	1	-	1

**Table 2:** Primer and probe sequences used in this study.

Primer	Sequence (5′→3′)	Position (nt) ^a^	Virus	Region
	**Real-Time RT-PCR**			
ZIKV835	TTGGTCATGATACTGCTGATTGC	835–857	ZIKV	Matrix
ZIKV911c	CCTTCCACAAAGTCCCTATTGC	911–890		Envelope
ZIKV860-FAM ^b^	CGGCATACAGCATCAGGTGCATAGGAG	860–886		Envelope
ZIKV1086	CCGCTGCCCAACACAAG	1086–1102	ZIKV	Envelope
ZIKV1162c	CCACTAACGTTCTTTTGCAGACAT	1162–1139		Envelope
ZIKV1107-FAM ^b^	AGCCTACCTTGACAAGCAGTCAGACACTCAA	1107–1137		Envelope
	**RT-PCR for Sequencing**			
ZIKENVF	GCTGGDGCRGACACHGGRACT	1643–1663	ZIKV	Envelope
ZIKENVR	RTCYACYGCCATYTGGRCTG	1989–2008		Envelope
D1	TCAATATGCTGAAACGCGCGAGAAACCG	132–159	DENV	Capsid/prM
D2	TTGCACCAACAGTCAATGTCTTCAGGTTC	614–642		Capsid/prM

^a.^ The nucleotide numbering corresponds to that of the published ZIKV sequence [GenBank: AY632535.2] or DENV sequence [GenBank: DQ863638.1]. ^b.^ Probes are labeled with 5′ 6-FAM and 3′ TAMRA-N.

## Supplementary Materials

The following are available online at https://www.mdpi.com/2076-0817/9/6/442/s1, Figure S1: Alignment of 331 bp ZIKV partial envelop sequences, Figure S2: Alignment of 395 bp DENV partial capsid/prM sequences.
