# Supplementary material for: Entomological Surveillance for Zika and Dengue Virus in Aedes Mosquitoes: Implications for Vector Control in Thailand
Source: Pathogens. 2020 Jun 4;9(6):442. doi: 10.3390/pathogens9060442 (PMC7350330; doi:10.3390/pathogens9060442)
Supplement: Supplementary file 1 [file pathogens-09-00442-s001.pdf]

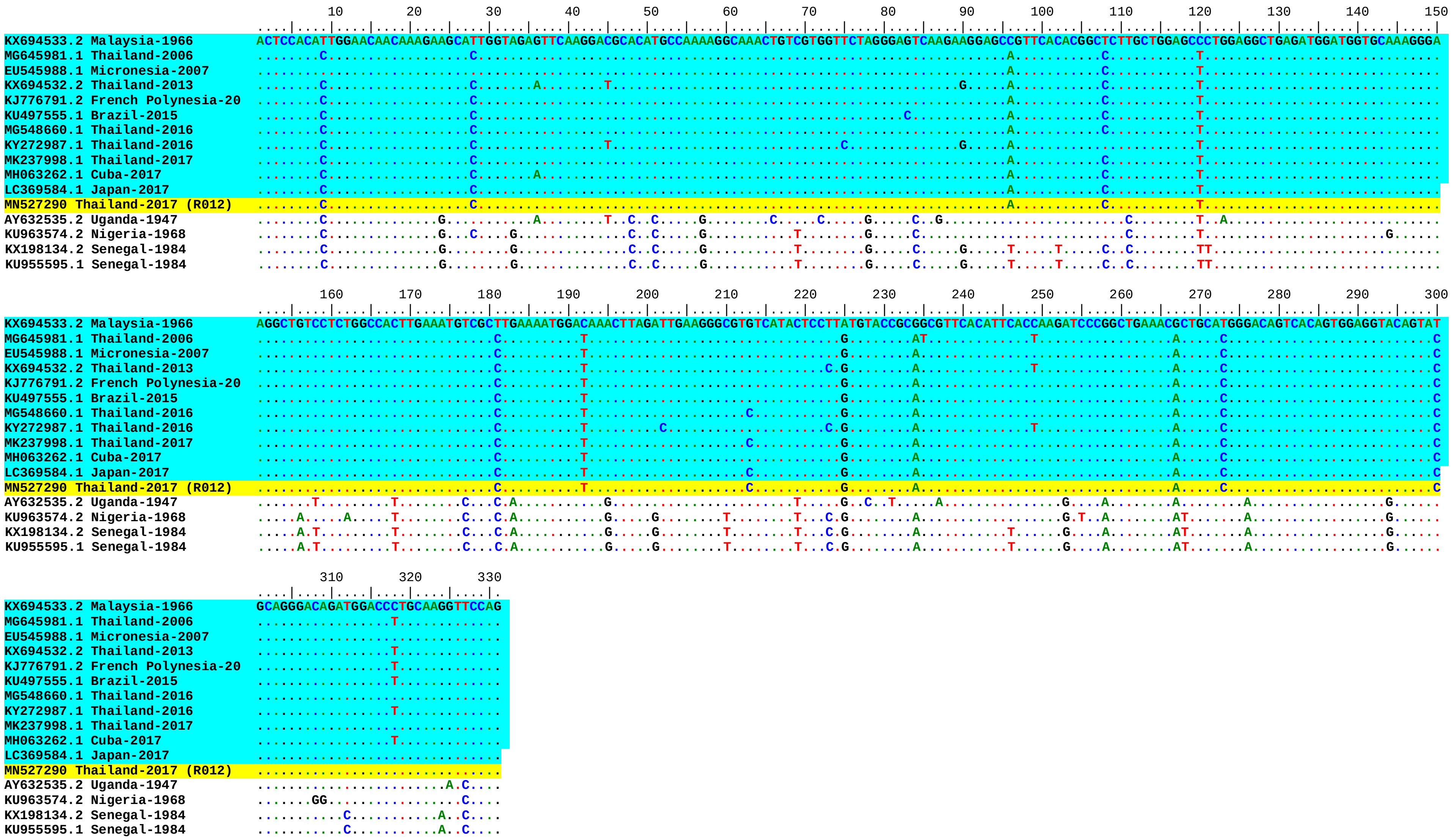

Figure S1 Alignment of 331 bp ZIKV partial envelop sequences. ZIKV sequence retrieved in this study is indicated with yellow highlight. Sequences of Asian lineage ZIKV are indicated with blue highlight. Sequences of African lineage ZIKV are with no highlight.

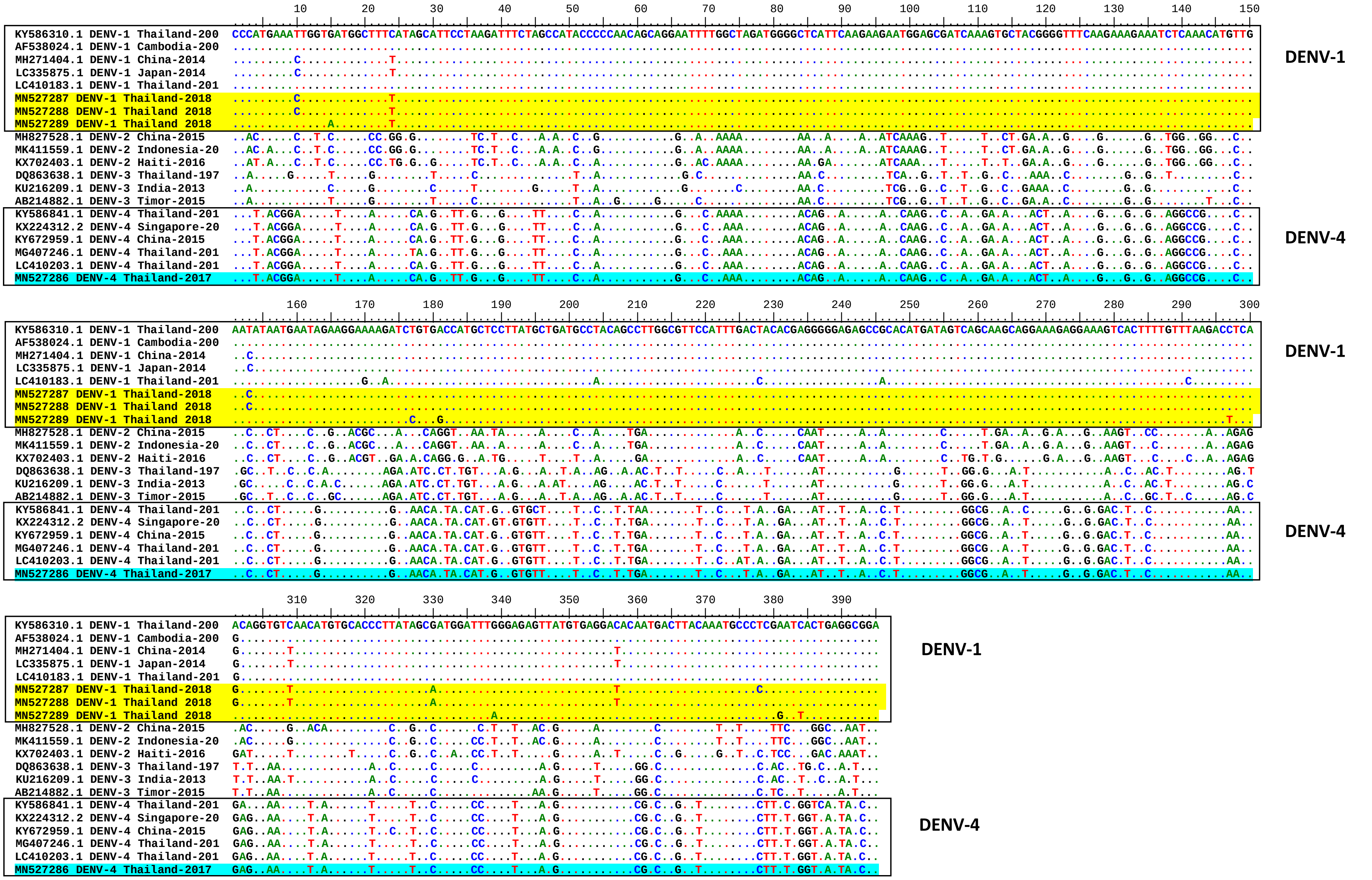

Figure S2 Alignment of 395 bp DENV partial capsid/prM sequences. DENV-1 and DENV-4 sequences retrieved in this study are indicated with yellow and blue highlight, respectively.
